# Fabrication of ultrathin CuO nanowires augmenting oriented attachment crystal growth directed self-assembly of Cu(OH)_2_ colloidal nanocrystals†

**DOI:** 10.1039/d0na00308e

**Published:** 2020-05-18

**Authors:** Gayani Pathiraja, Ryan Yarbrough, Hemali Rathnayake

**Affiliations:** Department of Nanoscience, Joint School of Nanoscience and Nanoengineering, University of North Carolina at Greensboro 2907 East Gate City Blvd Greensboro NC27401 USA hprathna@uncg.edu +1-336-285-2860

## Abstract

Augmenting the oriented attachment (OA) crystal growth phenomena, herein, we demonstrate fabrication of ultrathin CuO nanowires from self-assembled one-dimensional (1D) nanowires of Cu(OH)_2_ nanocrystals. A facile environmentally benign sol–gel approach, which utilizes base-catalyzed hydrolysis followed by directed self-assembly and crystal growth of nanocrystals, is developed to prepare Cu(OH)_2_ nanowires. The sol of Cu(OH)_2_ nanocrystals shows aggregative self-assembly guided by the OA crystal growth process to form ultrathin Cu(OH)_2_ nanowires, with an average length of 675 ± 4 nm and diameter of 6 ± 2 nm. The time-dependent UV-visible spectral traces, along with real-time imaging of nanocrystals self-assembly and growth under the transmission electron microscope, are evidenced the concept of the OA crystal growth directed self-assembly, yielding 1D colloidal nanoarrays of Cu(OH)_2_. The powder XRD traces collected during the self-assembly and crystal growth process reveal the directional aggregative crystal growth along the facet of [001], confirming the OA directed crystal growth and fusion of nanocrystals to yield 1D nanostructures. The gradual blue-shift in optical absorption maxima from 770 nm in initial precursor solution, to 670 nm for Cu(OH)_2_ nanocrystals sol, and finally to 647 nm for self-assembled 1D Cu(OH)_2_ nanowires have further evidenced the formation of Cu(OH)_2_ nanowires. Upon subjecting self-assembled Cu(OH)_2_ nanowires for post-annealing treatment, ultrathin CuO nanowires with average length of 7 ± 0.50 μm and diameter of 27 ± 2 nm is obtained in high purity. The experimental powder XRD patterns of Cu(OH)_2_ and CuO nanowires match the simulated XRD patterns, indexing the crystal unit cell structures to orthorhombic and monoclinic, respectively. The tailored narrow optical band gaps for Cu(OH)_2_ and CuO nanowires are found to be 1.51 eV and 1.10 eV. The theoretical band gap predicted for Cu(OH)_2_ nanowires is 1.52 eV and is in good agreement with its optical band gap, whereas theoretical band computed for CuO nanowires is 0.13 eV lower than from its optical band gap.

## Abstract 2

Augmenting the oriented attachment (OA) crystal growth phenomena, the fabrication of ultrathin CuO nanowires is demonstrated from self-assembled one-dimensional (1D) nanowires of Cu(OH)_2_ nanocrystals for the first time.

## Graphical abstract

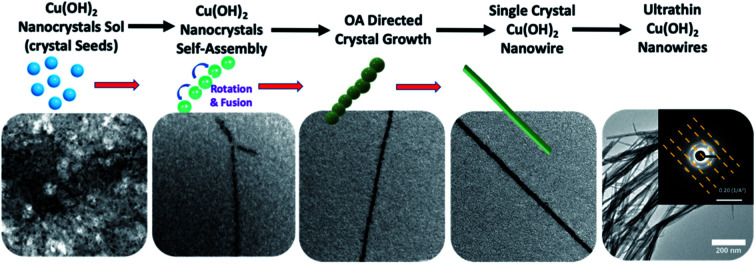

## Introduction

Inorganic functional materials designed at nanoscale with well-defined geometries, having unique size- and shape-dependent properties, are of significant interest compared to their bulk counterparts. Control over dimensions as well as composition of structures make it possible to tailor material properties for a specific application. For example, inorganic semiconductor nanocrystals exhibit a wide range of size-dependent superior properties for optics and electronics.^1–3^ Among all inorganic functional nanomaterials, the size-dependent unique characteristics of metal oxide nanostructures make them the most diverse class of materials, where their properties encompass almost all the fields of materials science and nanotechnology. Upon varying the metal type, a large diversity of binary, ternary, and mixed-oxide nanostructures has been synthesized^4–8^ and tailored their geometries at nanoscale. Up to date, many structural geometries with an electronic structure that exhibits metallic,^9,10^ semiconducting^9,11^ or insulating^12^ character have been prepared for a broad range of applications, such as for catalysis, nanoelectronics, sensing, optics, solar cells, medical diagnostics, drug delivery, cellular signalling, and nanomedicine.^13–20^

Up to date publications witness that new metal oxide nanostructures with superior electronic properties are reported almost on a daily basis. However, in-depth scientific understanding of metal oxide nanocrystals' growth mechanisms, kinetic rules of controlling size and morphology, and accompanying phase transformations is rarely explored.^21^ Among various synthetic approaches,^22–24^ wet chemical synthesis exhibits scientific and practical significance for understanding and controlling the size and shape at nanoscale *via* bottom-up crystal growth processes. But crystal growth kinetics are difficult to control as colloidal nanocrystals undergo rapid nucleation and growth, while interacting with surrounding matrix. The formation of microstructures from colloidal solution of nanocrystals is usually explained by Ostwald ripening (OR) theory.^21,25,26^ The OR mechanism has been widely used to explain the crystal growth of nanocrystals that produce particles with larger diameter, typically in the micro-meter size range. However, in some circumstances, the crystal growth of nanocrystals in the nano-meter regime was often unable to explain by the OR kinetics.^27–29^ At nanoscale, it has been evidenced that crystal growth rather dominates by an alternative mechanism, named oriented attachment (OA), in which nanocrystals self-assemble into a single crystal by sharing a common crystallographic orientation.^30,31^ The concept of “OA” was first introduced by Banfield *et al.* who studied the hydrolytic synthesis of TiO_2_ nanocrystals.^32^ Since then, this aggregation-based crystal growth concept has been attractive for constructing nanoscale materials.

As the OA process enables the self-assembly of primary nanocrystals by augmenting bottom-up fabrication processes, it can produce novel structures with versatile properties, different from those of the corresponding bulk materials. In particular, the OA process has demonstrated as an effective approach to prepare anisotropic nanostructures, where attachment of nanocrystal seeds always guides the self-assembly to one orientation, generating one-dimensional nanowires or nanorods.^33–35^ In the OA mechanism, the crystal growth rate is correlated exponentially to the surface energy. The crystal growth takes place along a specific crystal face, depending on the relative specific surface energies associated with the facets of the crystal.^36^ The differences of surface-energy at each face induce much faster growth of the higher surface energy planes and keep the lower surface energy planes as the facets of the product. For example, it has shown that anatase TiO_2_ nanocrystals formed one-dimensional necklace-shaped nanostructures by fusing nanocrystals along the [001] direction as a result of surface energy difference between the [001] and [101] faces, facilitating directional crystal growth of the OA mechanism.^32^ In another recent study, the formation of iron hydroxide particles directed by the OA mechanism was viewed in real time, evidencing nanocrystals' rotation and crystallographic orientation during the crystal growth.^37^ The OA was also demonstrated in the preparation of ZnO nanorods,^38^ MnO multipods,^39^ rare earth metal oxides nanoparticles^40^ and mixed oxides nanostructures with a wide variety of morphologies.^21^ Despite all these OA-directed synthesis of shape and size-controlled metal oxides and mixed oxide nanostructures with variety of morphologies,^21^ there are very few examples on the OA driven wet-chemical syntheses for constructing size-controlled metal oxide nanowires.^41,42^

Among wet-chemical synthesis methods, the sol–gel chemical route is one of widely applied techniques to prepare highly stoichiometric and high-quality (less defects) ultrafine metal oxide nanostructures at low temperature using environmentally benign conditions.^43–51^ Owing to its versatility, scalability, and solution processability, sol–gel route is adaptable to prepare metal oxide nanostructures, with better control over the crystal growth, morphology, and dimensionality. However, past studies are absent from the development of a reliable OA directed sol–gel-based self-assembly driven synthesis method, which is rapid, environmentally benign, and performed under ambient conditions. To the best of our knowledge, the existing OA directed crystal growth techniques are based on hydrothermal methods, which involve surfactants, medium to low temperatures, and longer reaction time (sometimes up to 2–4 days).^21^ Herein, we demonstrate, for the first time, preparation of ultrathin nanowires, utilizing a sol–gel-based OA directed self-assembly process. This novel self-assembly driven process provides in-depth understanding of the OA crystal growth process that can utilize to make dimensionality controlled anisotropic nanostructures. In particular, this is the first example of synthesizing ultrathin CuO nanowires from self-assembled Cu(OH)_2_ colloidal nanocrystals by a versatile OA directed bottom-up self-assembly method in water, followed by post-annealing at 300 °C. Previously, once, the oriented attachment crystal growth was demonstrated in the synthesis of shuttle-like CuO nanocrystals *via* a hydrothermal decomposition method from an aqueous solution of copper hydroxide without any surfactants at low temperature (120 °C).^52^ In general, CuO nanowires were prepared using most common physical processes, such as vapor phase evaporation^53,54^ and direct thermal oxidation.^55–60^ Although a few reports have been reported sol–gel chemical routes to make CuO nanowires,^61–66^ most of such synthesis developments were not successful in producing high aspect ratio CuO nanowires with high crystalline, and tailored optical and electronic properties. Therefore, the work described herein addresses the key challenge of producing high aspect ratio CuO nanowires *via* traditional wet-chemical synthesis routes. Moreover, the OA-directed sol–gel route developed here can widely apply to make variety of metal oxide nanowires in larger scale with better reproducibility, compared to the current sol–gel approaches, which follow either hydrothermal or solvothermal processes.

A facile base-catalysed aqueous sol–gel synthesis combined with a directed self-assembly of nanocrystals sol and their aggregative crystal growth at low temperature (5 °C) was first conducted to prepare self-assembled one-dimensional (1D) nanowires of Cu(OH)_2_ nanocrystals. The nucleation, colloidal self-assembly, and crystal growth process of nanocrystals were investigated by collecting time dependent UV-visible spectral traces and transmission electron microscopy (TEM) images. The time-dependent analyses revealed the OA guided crystal growth process for the formation of 1D nanostructures. The crystallinity and nanocrystal packing were evaluated from the powder X-ray diffractions, which support the aggregative crystal growth mechanism. Post-annealing of self-assembled Cu(OH)_2_ nanowires, ultrathin CuO nanowires (CuO NWs) were fabricated. The chemical composition analysed by X-ray photon spectroscopy (XPS) revealed the respective chemical formula of each nanomaterials. The size-controlled optical properties, along with optical and theoretical band gaps of these 1D nanomaterials were also studied and revealed the morphology-controlled electronic structure properties at nanoscale.

## Results and discussion

**Scheme 1:**
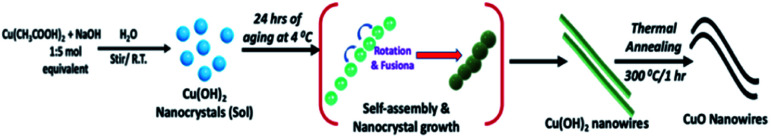
The chemistry and self-assembly process for the formation of Cu(OH)_2_ colloidal nanowires and CuO nanowires.

In our recent study, for the first time, augmenting both OR and OA processes, we developed a facile sol–gel approach, which combines solvent polarity driven self-assembly and solvothermal crystal growth process to prepare shape-controlled ZnO nanostructures.^51^ We demonstrated that the difference in polarity and surface adhesion property of an organic solvent controlled the nanocrystal growth where solvent molecules act as surfactants that adsorb onto surfaces of the growing crystallites.^51^ Thus, this sol–gel approach serves as a versatile wet-chemical synthesis path to create shape-controlled metal oxide nanostructures by modulating the surface energy of nanocrystals *via* selective adhesion of solvent molecules (act as surfactants). In contrast, the sol–gel approach developed in the present study established a foundation to prepare anisotropic nanostructures directed only by the OA crystal growth process in a surfactant free, an aqueous solution at room temperature. Specially, this novel wet-chemical synthesis approach that combines the sol–gel chemical process with directed self-assembly of colloidal nanocrystal serve as a facile route to prepare size- and shape-controlled metal oxide nanowires. The chemistry and self-assembly process to prepare ultrathin Cu(OH)_2_ and CuO nanowires is summarized in Scheme 1.

### Preparation and characterization of Cu(OH)_2_ colloidal nanowires

Sol–gel hydrolysis followed by colloidal self-assembly of nanocrystals was performed to prepare Cu(OH)_2_ nanowires. In a typical procedure, colloidal solution of Cu(OH)_2_ nanocrystals were prepared from the base-catalysed hydrolysis of aqueous solution of copper acetate (Cu(CH_3_COOH)_2_) in the presence of sodium hydroxide (NaOH) at room temperature. The molar ratio of the precursor to the base was maintained at 1 : 5. The nanocrystals formation, their self-assembly, and crystal growth to form Cu(OH)_2_ nanowires were monitored by acquiring time-dependent UV-visible spectra and TEM images.

**Fig. 1:**
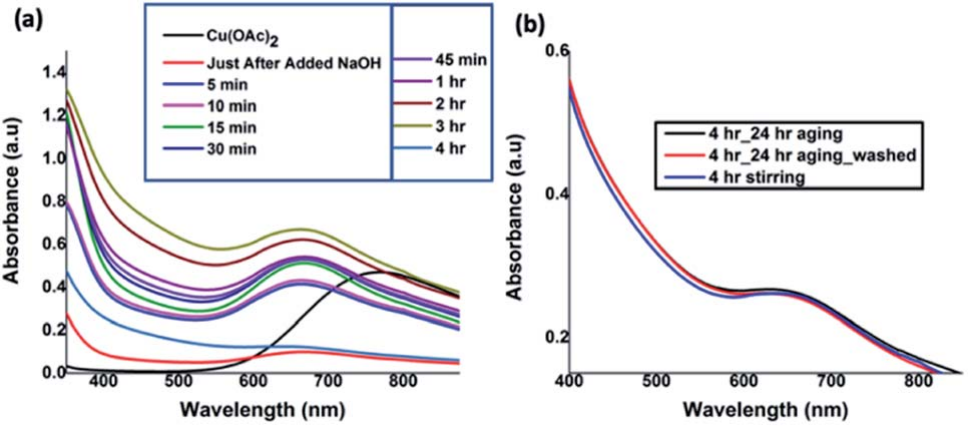
(a) UV-visible absorption spectra taken at different time intervals during the reaction (in water), and (b) comparison UV-visible spectra of after 4 h of reaction completion, followed by aging and centrifuged, washed, and re-disperse in water.

The UV-visible absorption spectra taken at nine different time intervals after the addition of NaOH revealed the progress of Cu(OH)_2_ nanocrystals formation and their assembly to form Cu(OH)_2_ colloidal nanowires. All the spectra were recorded in solution as either a clear solution or a suspension. As depicted in Fig. 1(a), initial absorption maxima for Cu^2+^ solution was observed at 770 nm. After the addition of NaOH drop-wise with the rate of 0.1 mL min^−1^ over 5 min time period, the absorption band at 770 nm shifted to 670 nm, evidencing the hydrolysis of Cu(OAc)_2_ to form Cu(OH)_2_ seeds (nanocrystals sol). With time, the maximum absorption was gradually shifted to blue region. The absorption maximum was unchanged for the reaction time intervals from 15 to 45 minutes and found to be stable at 667 nm and then exhibited a gradual shift to 647 nm after 4 h of reaction time. The gradual shift in the UV-visible absorption band from 770 nm for Cu (OAc)_2_ to 670 nm, and then to 667 nm, and finally to 647 nm confirms the hydrolysis, sol formation, and their self-assembly to 1D colloidal arrays. In order to avoid the ripening of nanocrystals while facilitating the nanocrystals self-assembly, the reaction was stopped after 4 h and subjected to aging 24 h at 5 °C. The UV-visible spectra (Fig. 1(b)), taken after 24 h aging, and the purified and re-dispersed Cu(OH)_2_ colloidal nanowires in water, exhibit no changes in the absorption maxima, evidencing there was no further ripening of nanocrystals, but rather facilitating the directed self-assembly of nanocrystal seeds to a specific orientation, generating 1D Cu(OH)_2_ nanowires.

**Fig. 2:**
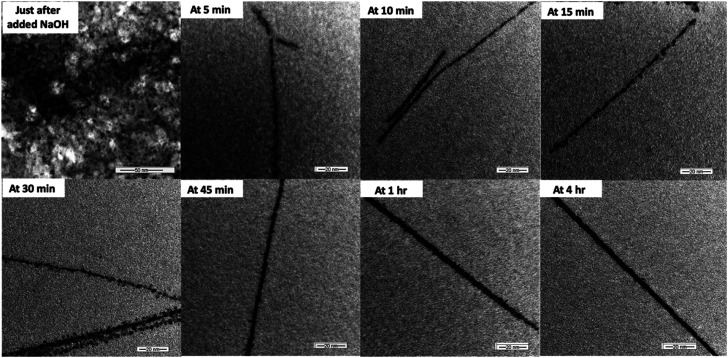
TEM images taken at different time intervals over 4 h just after the addition of NaOH solution.

To explore the driving force for colloidal nanowires formation that demonstrates the directed self-assembly and OA aggregative crystal growth, nucleation, nanocrystal formation, and their self-assembly process were monitored by imaging under TEM. As shown in Fig. 2, just after adding the base over 5 minutes of addition time, highly populated irregular shape small nanocrystals with the size ranged from 2–4 nm were observed. As revealed from the TEM images taken at 5 min of reaction time, nanocrystals started to self-assemble into one direction by connecting each other while leading the coalescence of nanocrystals in specific crystallographic orientation. Past studies showed that due to coalescence, a coherent crystallographic orientation eliminates the interfaces of nanocrystals, resulting in the reduction of surface energy, which drives the OA crystal growth.^21^ The similar self-assemblies of nanocrystals were observed in the past where the difference of surface energy at each crystal facet leads to the coalescence of primary particles in specific crystallographic orientation. For example, the one-dimension growth along the [001] crystal facet of TiO_2_ nanocrystals to form TiO_2_ nanorods^67^ and growth along the [111] facet to form PbSe nanowires were augmented in the literature.^68^ As the reaction proceeds, we observed formation of larger nanocrystals with rough and irregular surface features, by consuming initial nanocrystals as “building blocks”. Thus, it is clear that the size-controlled crystal-growth along one specific direction of nanocrystal's facet is guided by the spontaneous self-organization of adjacent particles, having the same crystallographic orientation to form one big elongated nanocrystal. The dimension analysis (Table S1†) at different reaction time intervals further supports the size-controlled crystal growth. After 15 minutes, there was no increased in the diameter of colloidal nanowires and the average diameter was found to be constant at 6 ± 2 nm throughout rest of the reaction time. Thus, the crystal growth was observed along the long axes of nanocrystals. The maximum average length of nanowires was reached up to 518 ± 2 nm at 4 h from the initial average length of 200 ± 2 nm at 5 minutes of reaction time, after addition of the base. These results further suggest that the nanowires formation follows the OA guided spontaneous self-assembly directed crystal growth process. After 45 minutes, the surface of colloidal nanowires tends to smooth, and with longer stirring times (1 h and 4 h), there was no indication of a significant increase in the diameter of nanowires. The effect of aging time on crystal growth and nanowires dimensionality was also investigated by aging 4 h stirred colloidal solutions for 6 h and 24 h (Table S2†). After 6 h of aging, we observed an increase in the diameter of nanowires by ∼1–2 nm with ∼120 nm increase in the length. However, after 24 h of aging, we observed a slight decrease in the average diameter, while maintaining the nanowires' length same as the average length of nanowires after 6 h of aging. After 24 h of aging, the reaction resulted fully grown ultrathin Cu(OH)_2_ nanowires with an average length of 675 ± 4 nm and diameter of 6 ± 2 nm. The TEM images (Fig. S1†) of fully grown Cu(OH)_2_ nanowires exhibit stacks of very smooth hair like wires.

**Fig. 3:**
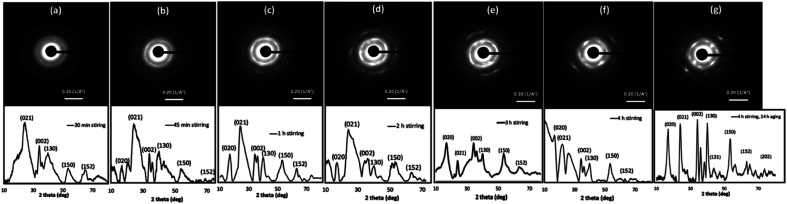
Time dependent SAED patterns along with powder XRD traces of Cu(OH)_2_ colloidal nanocrystals at the stirring time of: (a) 30 min, (b) 45 min, (c) 1 h, (d) 2 h, (e) 3 h, (f) 4 h, and (g) 4 h followed by 24 h aging.

The OA crystal growth process was further explored by studying the time-dependent nanocrystals growth and crystallinity of self-assembled colloidal nanostructures. The powder XRD traces were acquired for powder samples collected, washed, and dried from the reaction at each time interval over 4 h Fig. 3 represents the time dependent SAED patterns and powder XRD patterns. The SAED patterns collected over four-hour period exhibit gradual growth in the diffraction patterns, in which more pronounced ring patterns are visible with longer stirring time. For example, after 30 minutes stirring time, the SAED pattern shows few dot patterns with discontinuous ring pattern, whereas after 1 h of stirring time, the intensity of the dot pattern is rather pronounced, and the ring pattern is more visible and denser. The SAED patterns obtained for samples after stirring time of 3 h, 4 h, and 4 h followed by 24 h aging show clear trend in directional crystal growth where we observed well-resolved dense crystal dot pattern. These results suggest the growth of single crystalline pattern during the 24 h aging to form highly crystalline Cu(OH)_2_ nanowires.

The time-dependent powder XRD analysis also reveal the gradual coalescence, re-orientation, and growth of crystal facets over four-hour stirring time and during 24 h aging period. According to the past literature, the powder diffraction pattern of highly crystalline Cu(OH)_2_ nanowires exhibits six main diffraction planes; [020], [021], [002], [130], [150], and [152], indexing the crystal unit cell to orthorhombic.^69^ As depicted in Fig. 3, the powder XRD traces collected over four-hour period show gradual growth of crystalline facets, having above listed six main diffraction planes. At the initial stages of stirring time (15 min and 30 min), XRD spectra exhibit only [021], [002], and [130] as pronounced reflection planes (Fig. 3(a) and S2†). For example, the powder XRD pattern for the sample collected after 30 minutes of stirring time, shows three major Bragg's peaks for the reflection planes of [021], [002], and [130] along with broader less intense two Bragg's peaks for [150] and [152] reflection planes. With longer reaction periods, the [020] reflection plane is visible while other reflection planes are becoming more resolved, broadened, and pronounced. The XRD patterns from the 45 minutes and up show the growth of the [020] crystal diffraction plane, evidencing the formation of wires by fusing of nanocrystals along the [020] facet. The powder XRD patterns, collected at 1 h and after, exhibit well-resolved six main diffraction planes with some gradual broadening of diffraction peaks, evidencing the crystal growth. After 24 h of aging, the SAED pattern and the powder XRD trace exhibited single crystalline diffraction pattern (Fig. 3(g)), and matches with the simulated diffraction pattern, indexing the unit cell structure to orthorhombic (Fig. S3†). Thus, the final XRD pattern obtained for the sample collected after 4 h stirring followed by 24 h aging confirms the formation of 1D-Cu(OH)_2_ nanowires.

**Fig. 4:**
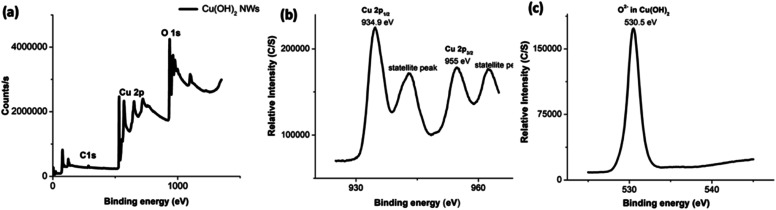
The XPS spectra of Cu(OH)_2_ nanowires; (a) the survey XPS spectrum, and the binding energy spectra of: (b) Cu 2p and (c) O 1s.

The composition and oxidation state of nanowires was elucidated by X-ray photoelectron spectroscopy (XPS) and supports the formation of highly pure Cu(OH)_2_ nanowires. The XPS survey spectrum depicted in Fig. 4(a) confirms the presence of Cu and O with the atomic ratio of 1 : 2 (Table S3†) indexing the nanowires composition to Cu(OH)_2_. Fig. 4(b) and (c) show the binding energy spectra for Cu 2p and O 1s respectively. The binding energy spectrum of Cu 2p exhibits two major peaks at 933.9 eV and 954.7 eV, which are characteristic to the binding energy states for Cu 2p_1/2_ and Cu 2p_3/2_ of Cu–O bonds in Cu(OH)_2_.^70^ The accompanied two less intense satellite peaks consistent with the presence of divalent Cu rather than Cu(0).^70^ With slightly positive binding energies of two main peaks for Cu 2p and the slightly larger FWHM, which is 6.05 eV (Table S3†), the composition of nanowires can be confirmed to hydroxide form. In the past studies, it has been reported that binding energies for Cu 2p_1/2_ and Cu 2p_3/2_ in Cu(OH)_2_ exhibit peaks at more positive binding energies by 1.2–1.5 eV with rather larger FWHM.^70,71^ As shown in Fig. 4(c), the presence of a well-resolved single peak for O 1s at 530.5 eV with a larger FWHM (4.47 eV) further supports the oxygen chemical bonding state of O^2−^ with the hydroxide form. The binding energy of O 1s peak for the oxide form is usually lower by ∼1.2 eV with very narrow FWHM (0.94 eV).

### Fabrication and characterization of CuO nanowires

**Fig. 5:**
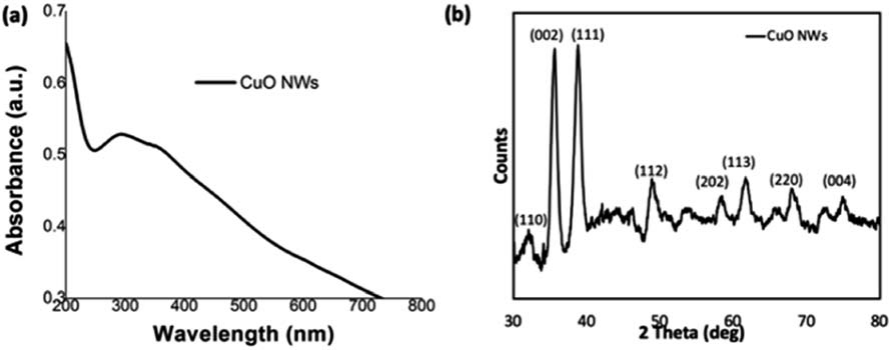
(a) Thin film UV-visible absorption spectrum, and (b) the powder XRD spectrum of CuO nanowires.

The CuO nanowires were fabricated from the Cu(OH)_2_ nanowires prepared from the sol–gel reaction of 4 h stirring followed by 24 h aging. Upon annealing the wet powder sample of Cu(OH)_2_ nanowires for an hour at 300 °C in a pre-heated furnace, the CuO nanowires were obtained in a considerably good yield. The fabrication of CuO nanowires in a thin film form was also conducted by annealing a drop-casted sample on Si wafer. The morphology and composition analysis (SEM, powder XRD, and XPS) of CuO nanowires were evaluated. As depicted in Fig. 5(a), the thin film UV-visible spectrum, obtained after annealing Cu(OH)_2_ nanowires for an hour at 300 °C, exhibits an absorption maximum at 292 nm, confirming the formation of copper oxide from its hydroxide.

The powder XRD traces collected for the annealed powder exhibits a characteristic diffraction pattern, which is different from the XRD pattern of Cu(OH)_2_ nanowires. The formation of CuO nanowires and its crystallinity reveals from the most dominant diffraction peaks at 2*θ* angle of 35.55° and 38.81°, which correspond to Bragg's diffraction planes of [002]/[−111], and [111], shown in Fig. 5(b). The peak intensity heights of [−111] and [111] reflections are comparable each other and evidence the favourable direction of wires' growth along the both facets of [−111] and [111] planes. The less intense diffraction peaks, which index to [110], [112], [202], [113], [220], and [004], supports the purity of CuO nanowires, indicating that Cu(OH)_2_ completely converted to CuO upon annealing. Comparing the experimental powder XRD pattern with the simulated XRD pattern, acquired from the crystallographic open data base for crystal structures of copper oxides; CuO and Cu_2_O, the chemical formula of the oxide form was identified. The simulated XRD pattern matched the experimental pattern, confirming the crystal structure to CuO, and indexes to monoclinic unit cell (Fig. S4†).

**Fig. 6:**
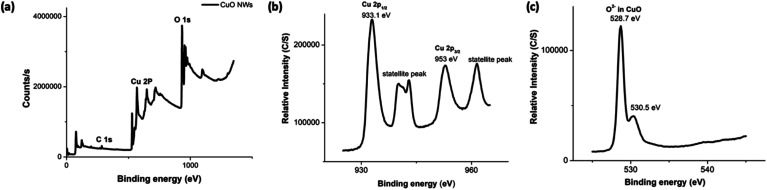
The XPS spectra (a–c) of CuO NWs; (a) the survey XPS spectrum, and the binding energy spectra of: (b) Cu 2p and (c) O 1s.

The composition and oxidation state of the CuO nanowires were elucidated by XPS measurements. The survey XPS spectrum in Fig. 6(a) evidences the coexistence of Cu and O with an atomic ratio of ∼1 : 1 for Cu and O, confirming the presence of only CuO form. Fig. 6(b) and (c) show high-resolution XPS spectra of Cu 2p, and O 1s, respectively. In Fig. 6(b), the Cu 2p XPS spectrum displayed two dominant peaks at 933.1 and 953.5 eV, accompanied by two less intense their respective satellite peaks, consistent with the presence of divalent Cu rather than Cu(0).^70,72,73^ The presence of a well-resolved single peak for O 1s at 528.7 eV, which is ∼1.8 eV lower compared to O 1s binding energy of Cu(OH)_2_ and confirms the oxygen chemical bonding state of O^2−^ in the oxide form. The binding energy of O 1s peak for the oxide form is usually lower by ∼1.2 eV with very narrow FWHM. Thus, smaller FWHM of 3.02 eV and 0.94 eV for Cu 2p and O 1s further supports the formation of highly pure CuO.

**Fig. 7:**
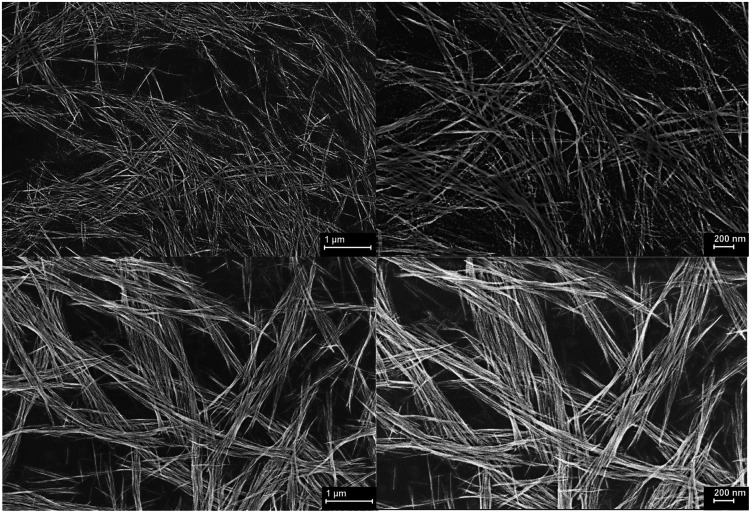
SEM images of CuO nanowires fabricated on Si-substrate.

The morphology of CuO nanowires was visualized from SEM and is shown in Fig. 7. The nanowires exhibit as stacks of very thin hair like wires with very smooth surface morphologies and appear to be continuous single crystalline wires. We observed in some cases, upon annealing the colloidal Cu(OH)_2_ wires, some wires were disassembled to nanocrystals as can be seen in Fig. 7(b). We speculate that during the annealing process, nanocrystal seeds were self-assembled to form colloidal nanocrystals arrays. But nanocrystal fusion and growth process were not completed to form single crystals (wires), yielding disassembled nanocrystals. During this temperature induced assembly and disassembly, the dimensional analysis (Table S4†) reveals the nanowire growth and further fusion of colloidal arrays of Cu(OH)_2_ to form CuO nanowires with an increase in the average length up to 7 μm and average diameter up to 25 nm. The increment in length is ∼10-fold compared to the average length of Cu(OH)_2_ nanowires and increment in average diameter is ∼4-fold compared to the average diameter of Cu(OH)_2_ nanowires. The dimensional growth of CuO nanowires during the annealing process suggest that there is a temperature dependent crystal growth and ripening to yield highly crystalline CuO nanowires. However, we have not investigated the effect of annealing temperature and duration on CuO nanowire dimensionality in this work.

### Electronic band structures of Cu(OH)_2_ and CuO nanowires

Correlating the single crystal unit cell structure to electronic structure of these ultrathin Cu(OH)_2_ and CuO nanowires enable us to discover their size and shape-controlled electronic properties. Thus, we estimated the optical band gaps and electronic band structures for Cu(OH)_2_ and CuO nanowires from their UV-thin film spectra, respectively. The optical band gap was calculated from the on-set of the UV-vis spectrum in each case.

**Fig. 8:**
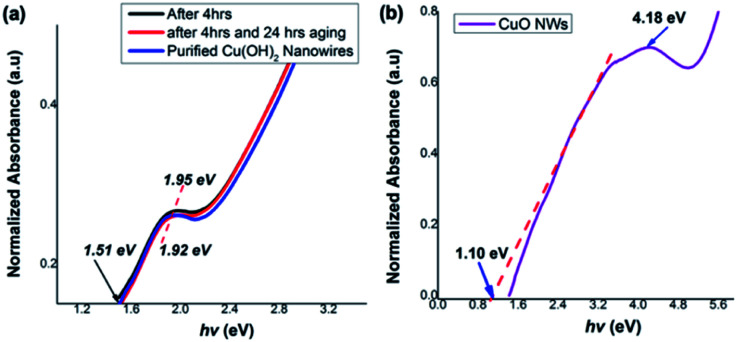
Thin film UV-visible spectra with respect to optical absorption energies for (a) Cu(OH)_2_ nanowires obtained after 4 h, 4 h followed by 24 h aging, and purified nanowires, (b) CuO nanowires.

As shown in Fig. 8(a), the photo-absorption energy (*hν*) of the absorption maximum for the Cu(OH)_2_ nanowires is found to be ranged from 1.95 eV for the colloidal nanoarrays formed after 4 h of stirring to 1.92 eV for the fully grown Cu(OH)_2_ nanowires, which were obtained after 4 h of stirring followed by 24 h of aging. The Cu(OH)_2_ nanowires' optical band gap, calculated from the UV on-set was 1.51, which is slightly narrower than the typical Cu(OH)_2_ bulk counterpart band gap of 1.97 eV.^74^ The optical band gap calculated for CuO nanowires from the UV-visible on-set was found to be 1.10 eV and exhibits the photo-absorption energy (*hν*) of 4.18 eV (Fig. 8(b)). The optical band gap estimated for CuO nanowires is slightly lower than the literature reported band gap of 1.2 eV.^65^ Rather narrow optical band gaps of Cu(OH)_2_ and CuO nanowires further confirms that nanowire's crystallinity, crystal growth facets, and crystal grain size lead to the effective narrow band gap of dimensionality controlled Cu(OH)_2_ and CuO nanowires.

**Fig. 9:**
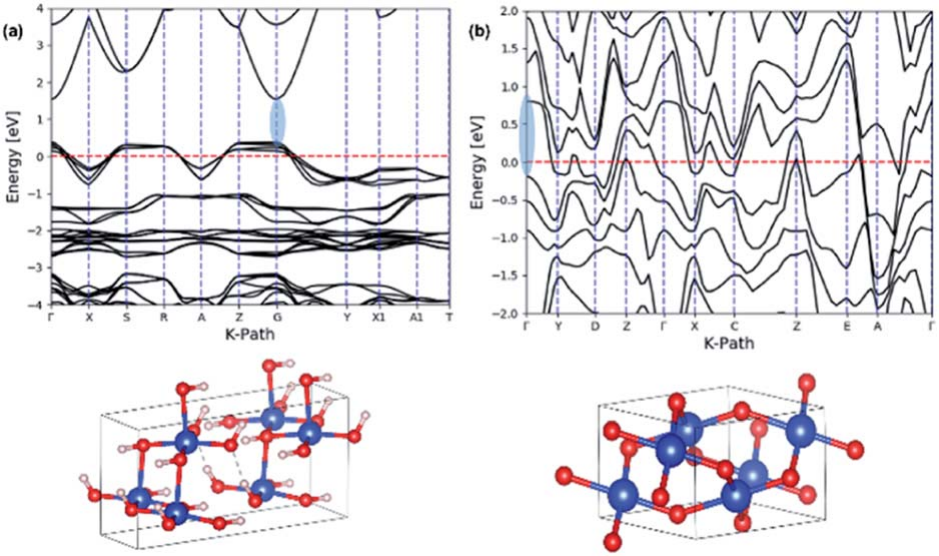
The calculated electronic band structures for: (a) Cu(OH)_2_ orthorhombic crystal unit cell structure, and (b) CuO monoclinic crystal unit cell. The energy of the valence-band maximum (VBM) was set to zero and used DFT and Hubbard U+DFT hybrid function for the calculation, respectively.

The theoretical band gaps for nanowires were also computed from their respective crystal unit cells, which were acquired from the Crystallographic Open Data (COD) base. The unit cell parameters for each crystal unit cell was determined by comparing the simulated XRD pattern of the extracted crystal unit cell structures with the experimental XRD traces of Cu(OH)_2_ and CuO nanowires, respectively. The theoretical electronic band structure was computed using the plane-wave form of Density Functional Theory (DFT) implemented in the open source Quantum ESPRESSO (QE) suite. Ultra-soft pseudopotentials created with low-density approximation (PBE and LDA) functions were used and were obtained from QE. The band gap for Cu(OH)_2_ was calculated using DFT whereas the band gap for CuO calculated using only DFT was underestimated, thus we used DFT+U, which uses an additional Hubbard potential (*U*) for each element to correct band overlap discrepancies. The Hubbard potentials for copper and oxygen to correct the large discrepancy were based on 7.5 eV and 0 eV, respectively. The potentials were applied to the 3d orbitals of the Cu atoms.^75,76^Fig. 9 shows the electronic band structures calculated for Cu(OH)_2_ nanowires using DFT and CuO nanowires using DFT+U, resulting in the band gaps of 1.52 eV for Cu(OH)_2_ nanowires and 0.98 eV for CuO nanowires. The theoretical band gap and the optical band gap of Cu(OH)_2_ are comparable each other and are in good agreement. However, the theoretical band gap calculated for CuO nanowires is 0.13 eV lower than the optical band gap of CuO nanowires. Thus, the narrow electronic band gaps obtained here in suggest that the synthesis method developed utilizing the size-controlled nanocrystal growth suits to tailor the band gap of metal oxide nanostructures.

## Experimental

### Materials

Copper(ii)acetate monohydrate (98–102.0% powder) was purchased from Alfa Aesar. Sodium hydroxide (98% purity) and ethanol (anhydrous, 99.5% purity) were obtained from Sigma Aldrich. They used as received without any purification otherwise specified.

### Characterization

The morphologies and dimensions of all nanomaterials were analysed using transmission electron microscopy (TEM Carl Zeiss Libra 120) at 120 keV and scanning electron microscopy (Zeiss Auriga FIB/FESEM). SEM was performed as prepared samples on the Si substrate without sputter coating for any additional conductive layer on it. The Atomic concentrations and binding energies of all the elements present in nanowires were obtained from X-ray photoelectron spectroscopy (XPS) using XPS-Escalab Xi+ Thermo Scientific electron spectrometer. The optical properties of samples were determined using ultraviolet-visible spectroscopy (Varian Cary 6000i). The powder XRD analysis was conducted using Cu Kα radiation (40 kV, 40 mA, *k* = 1.54 °A) with a speed of 90 s on the X-ray diffractometer (XRD, Agilent technologies Gemini).

### Typical sol–gel synthesis and self-assembly approach for Cu(OH)_2_ colloidal nanowires

To a 10 mL volumetric flask, copper(ii)acetate monohydrate (Cu(CH_3_COO)_2_·H_2_O, 40 mg, 0.2 mmol) was dissolved in 10 mL deionized (DI) water to make 0.02 M Cu^2+^ homogeneous stock solution. Separately, to a 10 mL volumetric flask, sodium hydroxide (NaOH, 40 mg, 1.0 mmol) was dissolved in 10 mL DI water to form a homogenous solution. To a 20 mL glass vial, the Cu^2+^ precursor solution (1.25 mL) taken from the stock solution was added. While it was stirring gently in a magnetic stirrer, the base, NaOH (1.25 mL) from the stock solution was added slowly at the rate of 0.25 mL min^−1^ over 5 minutes to yield a blue colour suspension. Then the reaction was capped and stirred for 4 hours at room temperature (24 °C) while monitoring the reaction progress at different time intervals. With the reaction proceeds, initial blue colour was changed to bluish green over 4 h period. The suspension prepared in this manner was kept in the refrigerator at 5 °C for 24 hours aging. The colour of the reaction mixture was changed from bluish green to green during the aging process. The green suspension was centrifuged and washed with DI water three times to remove salts and leftover starting materials. Without air drying, the wet green solid was resuspended in DI water (∼1 mL) and transferred to a glass vial. The UV-visible spectrum for the final products was obtained in DI water. The UV-visible spectra of Cu(OH)_2_ nanowires while stirring at different time intervals were obtained in the solution without washing. The washed suspension was drop casted on a carbon coated TEM grid, dried 1 hour in the fume hood and imaged under TEM. The thin film UV-visible spectra were obtained after centrifuging followed by washing with DI water and then resuspending the wet green solid in ethanol (∼1 mL) and drop casted on a cleaned quartz plate. The thin film samples prepared in this manner were dried in the fume hood. The powder XRD spectra of Cu(OH)_2_ NWs were obtained for the glass fiber coated with washed wet green suspension and dried completely in the fume hood. The re-suspended product was drop casted on a cleaned Si substrate and XPS spectra were performed after dried it completely in the fume hood.

### Fabrication of CuO nanowires from Cu(OH)_2_ colloidal nanowires

The CuO nanowires were fabricated from the Cu(OH)_2_ nanowires prepared by the sol–gel approached described above. In a typical fabrication process, the Cu(OH)_2_ nanowires were resuspended in DI water (∼1 mL) in vial to yield a wet paste. The furnace was preheated to 300 °C and the sample in the glass vial was annealed at 300 °C for one hour to yield a black powder of CuO nanowires (∼1.5 mg). The annealing process also conducted for a drop-casted sample on Si wafer and performed the morphology and composition analysis (SEM and XPS) along with the sample annealed on the Si wafer. The powder XRD spectra of CuO NWs were obtained for the glass fiber coated with washed wet green suspension and annealed at 300 °C for one hour in a preheated furnace. The glass fibre alone was annealed at 300 °C for one hour and subtracted from the sample data for the accurate results. The UV-visible spectrum was obtained for the black powder after resuspending in DI water (∼1 mL) with sonication (as necessary). The thin film UV-visible spectra were obtained after centrifuging followed by washing with DI water and then resuspending the wet green solid in ethanol (∼1 mL), drop casted on a cleaned quartz plate and annealed at 300 °C for one hour in a preheated furnace.

### The method used for the theoretical band structure calculation

The theoretical band gap and the respective band structure were computed using the open source Quantum ESPRESSO (QE) suite. QE uses plane-waves to find solutions to a specific density function theory (DFT) setup. The computations were done using ultra-soft pseudopotentials created with PBE and LDA functions. Based on an optimization calculation the global cut-off energy for the calculation was set at 100 Ry. This value was optimized using the pseudopotentials used in the computation. The integration over the Brillouin zone was done using a Gauss smearing method with a smearing parameter of 0.1 Ry. The CIF files of orthorhombic unit cell structure (COD# 9007849) of Cu(OH)_2_ and monoclinic unit cell structure of CuO were used for the computations. The computations for prediction of CuO band structure were run using a kinetic energy cut-off set to 70 Ry; this value was optimized using the pseudopotentials. The integration over the Brillouin zone was done using a Fermi–Dirac method using a smearing parameter of 0.001 Ry based on literature.^77^ The visualization software VESTA was used to compute the theoretical XRD from the CIF files. The experimental XRD was matched with the simulated XRD to index each unit cell's unit cell parameters.

## Conclusions

In summary, a facile environmentally benign base-catalyzed sol–gel approach, augmenting oriented attachment crystal growth directed self-assembly of colloidal nanocrystals, allow us to make ultrathin, highly crystalline 1D nanowires of Cu(OH)_2_ and CuO with high purity in large scale. The Cu(OH)_2_ nanowires formation demonstrates the oriented attachment crystal growth mechanism for the first time. The OA directed crystal growth process is confirmed from time-dependent studies of nanocrystals formation, their growth, and self-assembly for the nanoarray formation by imaging under TEM and acquiring powder XRD and SAED patterns. The time-dependent powder XRD traces evidence the OA directed crystal growth along the facet of [001] reflection plane. The purified Cu(OH)_2_ nanowires confirms its crystallinity and packing pattern, indexing the crystal unit cell to orthorhombic. The compositional analysis conducted using XPS confirms the oxidation states of Cu and O and the chemical formula from the atomic composition, supporting the formation of highly pure Cu(OH)_2_ nanowires. The CuO nanowires, fabricated upon annealing ultrathin Cu(OH)_2_ nanowires, exhibit single crystal nanowire growth along the facets of [−111] and [111] reflection planes, extending the nanowire length in 10-fold compared to the original nanowire length of Cu(OH)_2_ nanowires. The experimental powder XRD pattern of CuO nanowires match the simulated XRD pattern, indexing the crystal unit cell structure monoclinic. The compositional analysis performed using XPS confirm the Cu(ii) oxidation state with atomic composition ration of Cu to O 1 : 1, yielding high purity CuO. The optical band gaps for Cu(OH)_2_ and CuO nanowires are found to be 1.51 eV and 1.10 eV. The theoretical band gap predicted for Cu(OH)_2_ nanowires is 1.52 eV and is in good agreement with its optical band gap. However, the theoretical band gap computed for CuO nanowires is 0.13 eV lower than from its optical band gap. The OA-directed crystal growth approach demonstrates herein provide dimensionality controlled ultrathin 1D nanostructures, resulting in narrow band gaps with tailored electronic structures. Thus, the OA crystal growth directed sol–gel synthesis method developed in this work offers a promising guide to prepare size-and shape-controlled metal oxide nanostructures with tailored electronic properties.

## Conflicts of interest

There are no conflicts to declare.

## Supplementary Material

NA-002-D0NA00308E-s001
