# Supplementary material for: Fabrication of ultrathin CuO nanowires augmenting oriented attachment crystal growth directed self-assembly of Cu(OH)2 colloidal nanocrystals
Source: Nanoscale Adv. 2020 May 18;2(7):2897–906. doi: 10.1039/d0na00308e (PMC9419473; doi:10.1039/d0na00308e)
Supplement: NA-002-D0NA00308E-s001 [file NA-002-D0NA00308E-s001.pdf]

Electronic Supplementary Material (ESI) for Nanoscale Advances.
This journal is © The Royal Society of Chemistry 2020

Supporting Information

Fabrication of Ultrathin CuO Nanowires Augmenting Oriented Attachment Crystal Growth Directed Self-Assembly of $Cu(OH)_2$ Colloidal Nanocrystals

Gayani Pathiraja,[a] Ryan Yarbrough, [a] and Hemali Rathnayake*[a]

*[a]Department of Nanoscience, Joint School of Nanoscience and Nanoengineering, University of North Carolina at Greensboro, Greensboro NC 27401

Table S1: The dimension analysis of $Cu(OH)_2$ nanowires at different stirring time intervals.

Stirring time	Average Length (nm)± SD	Average Diameter (nm) ± SD
5 min	201.9±13.0	2.4± 0.4
10 min	222.2±17.1	2.4± 0.4
15 min	285.4±28.9	5.2± 1.2
30 min	352.1±26.7	5.5± 1.2
45 min	440.6±24.5	5.1± 0.7
1 h	457.3±27.0	5.0± 0.9
2 h	480.9±31.4	5.0± 1.0
3 h	499.6±25.5	5.9± 0.9
4 h	518.2±24.7	5.0± 1.1
5 h	550.7±22.6	5.0± 1.0
6 h	545.6±16.8	5.3± 1.6

Note: Average lengths of nanowires were calculated by taking nanowires with maximum lengths and nanowires with minimum lengths, thus SD represents the deviation of nanowires lengths.

Table S2: Length and diameter of $Cu(OH)_2$ NWs with respect to different aging time after 4hrs of stirring.

Trial #	Aging time [h]	Average Length (nm) ± SD	Average Diameter (nm) ± SD
1	6	658.7±32.6	6.4± 2.1
2	6	659.3±72.9	7.2± 2.3
3	6	685.7±48.5	7.1± 2.2
4	24	680.0±43.9	5.1± 2.0
5	24	676.9±86.4	5.5± 1.5
6	24	665.9±66.0	5.2± 2.1

Note: Average lengths of nanowires were calculated by taking nanowires with maximum lengths and nanowires with minimum lengths, thus SD represents the deviation of nanowires lengths.

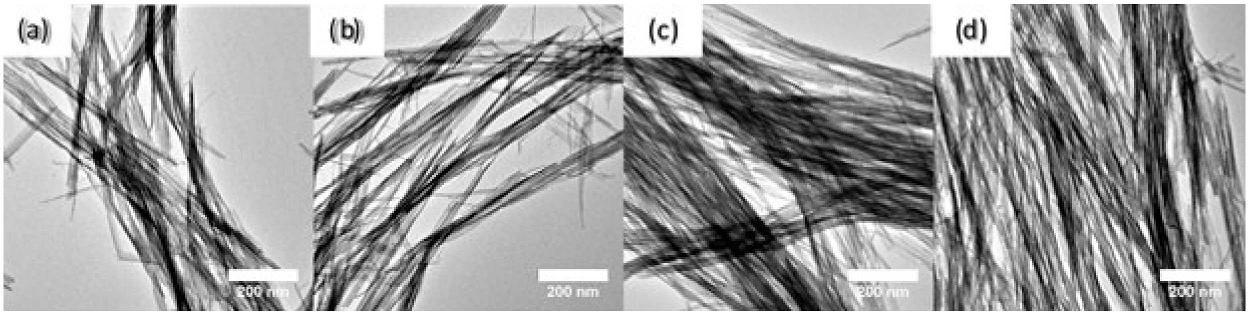

Figure S1: TEM images of fully grown $Cu(OH)_2$ nanowires at different stirring time intervals followed by different aging time intervals (a) 1 hr stirring followed by 6 hrs aging (b) 1 hr stirring followed by 24 hrs aging (c) 4 hrs stirring followed by 6 hrs aging (d) 4 hrs stirring followed by 24 hrs aging.

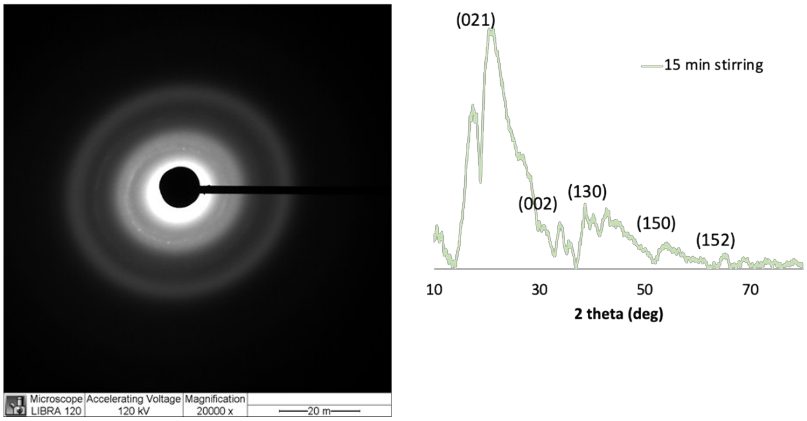

Figure S2: Time dependent SAED pattern along with powder XRD traces of $Cu(OH)_2$ colloidal nanocrystals at the stirring time of 15 min.

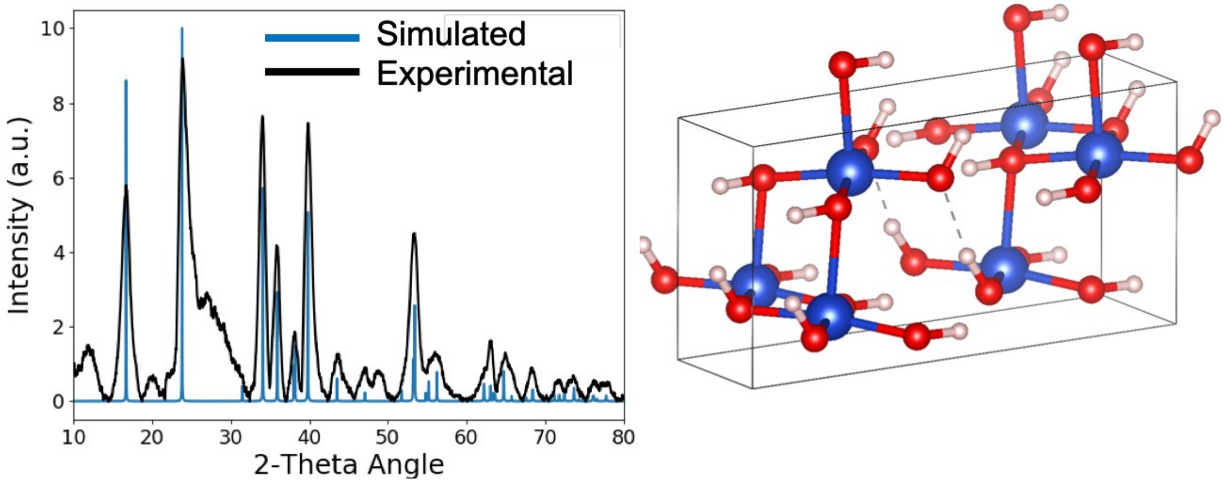

Figure S3: The simulated and experimental powder XRD patterns of $Cu(OH)_2$ nanowires and it's Orthorhombic crystal unit cell obtained from the Crystallographic Open Database (COD #9007849)

Table S3: XPS analysis of before annealed and after annealed NWs

Sample	Peak	Position BE (eV)±0.10 eV	FWHM (eV) ±0.20 eV	Atomic Con. (%)
$Cu(OH)_2$ NWs	Cu $2p_{1/2}$; Cu $2p_{3/2}$	934.9, 955.0	6.05	31.82
	O 1S	530.5	4.47	62.54
CuO NWs	Cu $2p_{1/2}$; Cu $2p_{3/2}$	933.1, 953.0	3.02	40.5
	O 1S	528.7, 530.5	0.94	42.96

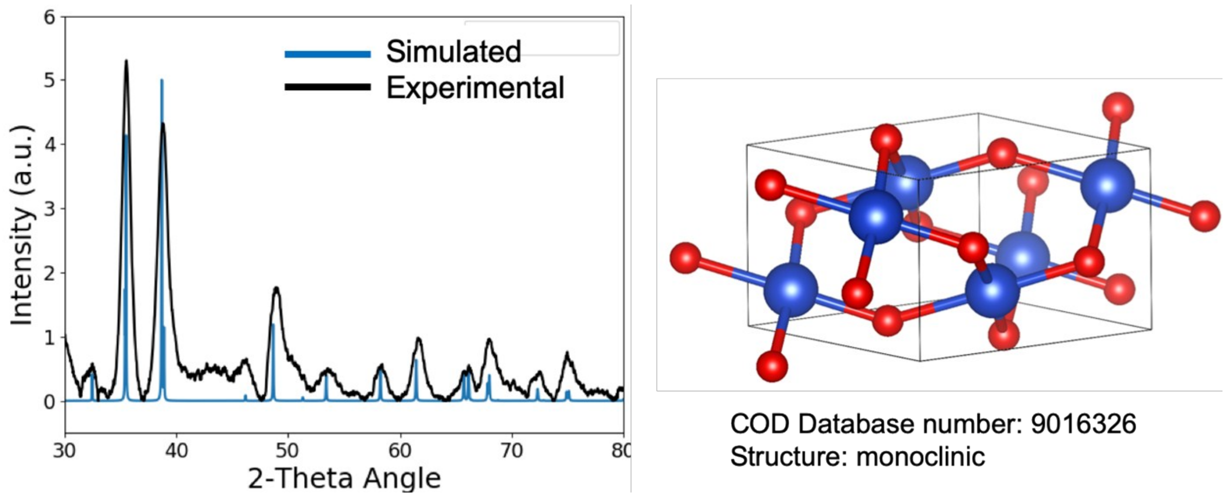

Figure S4: The simulated and experimental powder XRD patterns of CuO nanowires and it's Monoclinic crystal unit cell obtained from the Crystallographic Open Database (COD #9016326)

Table S4: Length and diameter of CuO NWs produced upon annealing $Cu(OH)_2$ nanowires, which were made at 4 hours stirring followed by 6 hours and 24 hours aging respectively. The powder samples of $Cu(OH)_2$ nanowires were annealed at 300 °C, an hour.

Trial	Aging time [h]	Average Length (µm) ± SD	Average Diameter (nm) ± SD
1	6	5.6±0.4	27.5± 6.0
2	6	5.5±0.3	31.6± 10.7
3	6	5.4±0.3	24.6± 6.9
4	24	7.7±0.4	29.4± 4.4
5	24	7.1±0.3	24.2± 4.4
6	24	7.5±0.3	26.0± 8.6

Note: Average lengths of nanowires were calculated by taking nanowires with maximum lengths and nanowires with minimum lengths. SD represents the deviation of nanowires lengths.
